# Simulation analysis and experimental study on cylinder grading of maize seeds based on discrete element method: A study on cylindrical grading of maize seeds

**DOI:** 10.1371/journal.pone.0335017

**Published:** 2025-10-23

**Authors:** Minji Liu, Jiannan Wang, Zhaoyan You, Chenyang Sun, Ni Wang, Jianchun Yan, Hai Wei, Huijuan Zhang, Huanxiong Xie

**Affiliations:** 1 Nanjing Institute of Agricultural Mechanization, Ministry of Agriculture and Rural Affairs, Nanjing, Jiangsu, China; 2 Jiangsu Academy of Agricultural Sciences, Nanjing, Jiangsu, China; NED University of Engineering and Technology, PAKISTAN

## Abstract

A cylindrical grader is an important piece of equipment used to grade maize seeds. However, the motion and distribution patterns of seeds within the cylindrical grading process remain poorly understood, leading to a heavy reliance on empirical adjustments of operational parameters during grading. This results in issues such as low grading efficiency, unstable operational performance, and failure to meet practical production requirements. To investigate the motion and distribution patterns of maize seeds during cylindrical grading, key simulation parameters characterizing the maize seeds and grading cylinders were experimentally determined. Discrete element models of maize seeds and the grading cylinder were subsequently developed using EDEM 2018 software. Variations in seed motion velocity and the coefficient of variation (CV) along the circumferential and axial directions were analyzed under different operational parameters, including cylinder rotational speed, inclination angle, and feeding rate. Discrete element simulations combined with orthogonal experiments revealed that the order of influence of these factors on the grading qualification rate was as follows: inclination angle > rotational speed > feeding rate. The results of the interaction analysis showed that the interaction between the inclination angle and rotational speed significantly affected the grading qualification rate, while the interactions among the other factors had no significant effect. The optimized parameter combination was identified as a rotational speed of 47.08 r/min, an inclination angle of 0.52°‌, and a feeding rate of ‌303.07 g/s‌, achieving a theoretical grading qualification rate of 97.24%. Validation experiments conducted with this optimal combination yielded a practical grading qualification rate of ‌93.83%‌, with the relative error between the experimental and predicted values below 4%‌. These results confirm the validity of discrete element simulations for analyzing maize seed motion dynamics and provide a valuable reference for further research in this field.

## Introduction

Maize is a crucial grain crop in China, playing a pivotal role in ensuring national food security. In 2024, China’s maize production reached 295 million tons [1], marking an increase of 6.1 million tons from the previous year and accounting for over 40% of the total grain output. The corresponding demand for seeds remains substantial. Seed grading is a critical pre-sowing process that produces uniform and high-quality seeds, facilitates mechanized sowing, and improves planting efficiency [2] and yield. Cylinder graders are widely used for maize seed grading due to their simple structure, precise screening, operational reliability, and ease of cleaning [3]. However, current parameter adjustments rely heavily on empirical methods, leading to challenges such as low grading efficiency and unstable performance.

Numerous scholars worldwide have conducted research on cylinder grading technology. Hu et al. [2007] developed a cylindrical seed grading device, detailing its basic structure, operating principles, key component designs, and main operational parameters [4]. Yang et al. [2022] created a cylindrical grader, elaborating on its operating logic, critical parameters, technical specifications, process requirements, application scope, and innovative designs for grading and cleaning components [5]. Fu et al. [2023] designed a cylindrical grading device for *Camellia* oleifera fruits by incorporating an auxiliary spiral auger to address suboptimal operational quality in existing grading equipment. They conducted 3D modeling of the grading device and performed simulation analyses to investigate the influence of key operational parameters on grading quality, ultimately identifying the optimal parameter combination [6]. Sun [2019] conducted force and motion analyses of garlic seeds within a grading cylinder to identify key factors affecting operational quality and their value ranges. A grading test bench was subsequently constructed for experimental research, through which the optimal parameter combination was determined [7]. Lv et al. [2019] developed a peanut cleaning and grading machine that employs impurity-removal and grading technologies based on the gravity and morphological characteristics of peanut pods. Post-processing results demonstrated that the system effectively eliminates both organic and inorganic impurities and achieves dimensional categorization of peanut pods [8]. Li et al. [2020] designed a compound cylindrical sorting device with curved grid screens for oats to address the low cleaning efficiency of oat-processing equipment. The study conducted theoretical analyses of the sorting principles and the dynamic force characteristics of the bouncing plate and baffle screens during operation [9]. Wang et al. [2020] addressed challenges posed by significant agronomic morphological variation and complex physical characteristics during mechanized Chinese cabbage seed harvesting. They developed a specialized sorting apparatus comprising an inward-flow cylindrical sieve and a cross-flow impurity extraction fan. A systematic theoretical analysis was conducted to elucidate the fundamental mechanisms of seed separation and cleaning [10]. Ashkiki et al. [2019] implemented a dual-cylinder screening system for municipal solid waste classification, investigating the impact of feeding rate, screen aperture blockage, and seasonal variations in waste composition on operational efficiency. Mathematical models were established to quantify correlations between these variables and separation performance metrics [11]. Stessel et al. [1996] established a computer model for rotary cleaning screens and designed testing procedures, revealing how material stratification, sieving duration, and bed layer slippage, affected by rotational speed, influence operational quality [12]. Nati et al. [2015] significantly improved the operational quality and efficiency of a cylindrical sieve classifier through experimental adjustment of parameter combinations, effectively removing large particles from pig feed to enhance feed quality [13].

In summary, current research has primarily focused on the design, testing, and analysis of key operational parameters affecting the grading quality of cylindrical graders, with an emphasis on equipment-related factors. However, during cylindrical grading, particle motion and distribution patterns represent another crucial factor influencing operational quality. Relevant studies—particularly those targeting maize seeds—remain limited. The cylindrical grading of maize seeds is a complex process involving competing mechanisms of particle mixing and separation within the cylinder, the underlying principles of which are still not fully understood.

This study investigates the cylindrical grading of maize seeds using the Discrete Element Method (DEM) to simulate the operational process, examine seed motion and distribution patterns under various conditions, and conduct validation experiments. This research contributes to improving the operational quality of cylindrical graders and provides valuable insights for related studies.

## Discrete element model and parameter settings

### Maize seed model

To establish a discrete element model for maize seeds, simulation-relevant characteristic parameters of the seeds were measured and obtained [14, 15]. The determination of discrete element parameters for granular materials is a well-established and widely used method, with extensive literature providing detailed reports on this subject. Ten maize seeds were randomly selected, and their original dimensions in the width and length directions were recorded. An extrusion test of the maize seeds was carried out using a electronic universal testing machine. The Poisson’s ratio was obtained by testing the dimensional change of a maize seed at the cracking limit in the width and length directions before and after pressure. When measuring the elastic modulus, the maize sample was placed on the flat plate of a universal testing machine. The loading speed and loading time were set to 15 mm/min and 5 seconds respectively. A probe was used to apply load along the thickness direction for 5 seconds before stopping the machine. The load and displacement data during the seed compression test were obtained using the software’s post-processing module. The ratio of the maximum compressive stress to the corresponding linear strain represents the elastic modulus. The shear modulus can be calculated using the elastic modulus and Poisson’s ratio. The methods for determining the static and dynamic friction coefficients were similar. The maize sample was placed on an inclined plane. When the inclination angle increased to the point where the surface object just began to show a tendency to slide, this angle was recorded as the static friction angle of the object on the inclined plane. Using this principle and method, the static friction coefficient of maize seeds was determined. When the inclined plane was slowly raised until the maize seed started moving downward, the angle between the inclined plane and the horizontal surface at that moment was identified as the dynamic friction angle, from which the dynamic friction coefficient of maize seeds was calculated. The inclined plane method is a common technique for measuring the restitution coefficient. Maize seeds were released from a specific height to free-fall, collided with the inclined plane, and rebounded in a parabolic trajectory. By measuring the corresponding horizontal displacements when the maize seeds were dropped from two different heights, the pre-impact and post-impact velocities relative to the normal direction of the inclined plane were calculated respectively, thereby determining the magnitude of the restitution coefficient. The results are summarized in S1 Table.

Twenty maize seeds were randomly selected [16] and their length (*L*), width (*W*), and thickness (*T*) were measured using a vernier caliper. The results are summarized in S2 Table.

**Fig 1:**
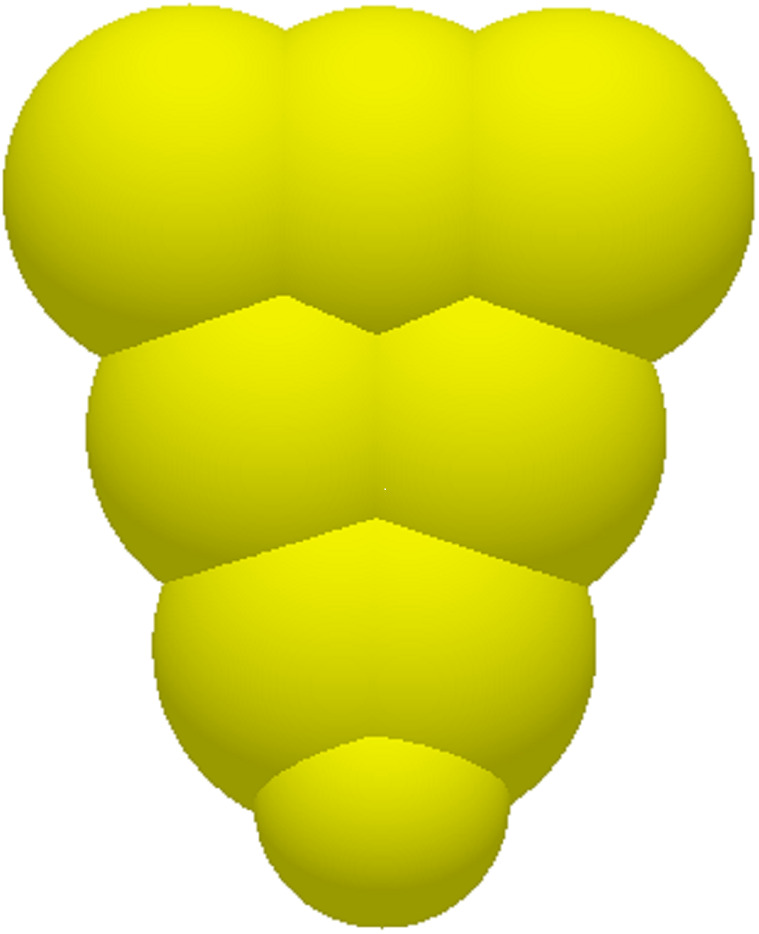
Simulation model of maize seed.

A maize seed model was constructed by aggregating multiple spherical particles to approximate their actual morphology [17,18]. The simulation model for the maize seeds is illustrated in Fig 1.

### Grading cylinder model

The grading cylinder was configured with a diameter of 450 mm and a length of 1500 mm, incorporating sieve apertures with an 8 mm diameter. Standard carbon steel was used as the cylindrical material, and the simulation-related parameters are listed in S3 Table.

**Fig 2:**
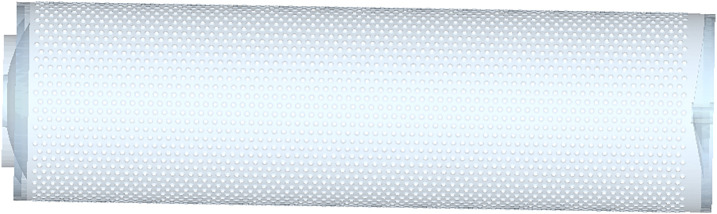
Simulation model of grading cylinder.

The geometric model of the graded cylinder was constructed based on the parameters specified in‌ S1 Table‌ and‌ S3 Table using ‌Autodesk Inventor 2016‌. The model was then exported in ‌STEP format‌ and imported into ‌the EDEM 2018 software‌ for discrete element method simulation. A 3D representation of the cylinder model is illustrated in ‌Fig 2‌.

### Stacking angle comparison test

The stacking angle is an important parameter to characterize the flow characteristics and friction characteristics of granular materials. In this study, physical tests were carried out, and the average value of stacking angle obtained by repeating 5 tests was 22.93°. At the same time, a stacking angle test model was established, and simulation tests were performed. Parameter intervals for the simulation were selected according to the measured parameters, as shown in S1 Table. Three factors affecting the stacking angle significantly were determined using the Plackett–Burman test: the static and dynamic friction coefficients of maize–maize and restitution coefficient of maize–maize. These factors were studied using the steepest climbing test to obtain a range of values for each factor. The average value was selected for other non-significant factors, that is, the Poisson’s ratio of maize was 0.4, the shear modulus of maize was 1.34 × 10^8^ Pa, the static friction coefficient of maize–steel plate was 0.385, the dynamic friction coefficient of maize–steel plate was 0.051, and the restitution coefficient of maize–steel plate was 0.541. A quadratic polynomial regression model of the stacking angle considering significant factors was obtained using the Box–Behnken test. Taking the actual stacking angle of 22.93° as the optimization objective, the optimal combination was formed: the static and dynamic friction coefficients were 0.527 and 0.042, respectively, and the restitution coefficient was 0.318.

**Fig 3:**
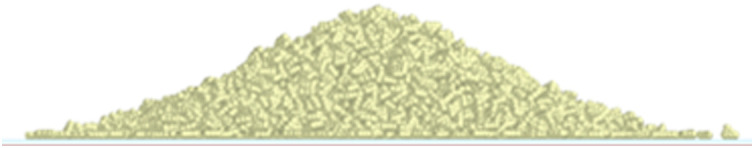
Simulated test.

**Fig 4:**
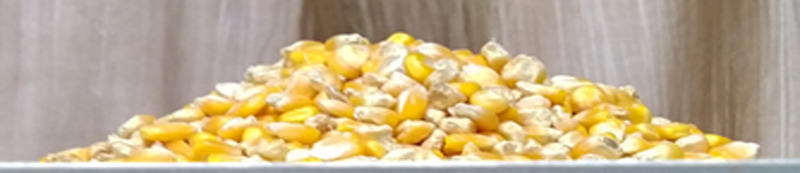
Physical test.

In order to verify the accuracy and effectiveness of the discrete element parameters of maize seeds, the above parameters were used for edem simulation test. The stacking angle obtained was 23.72 °. The relative error between the simulation value and the measured value was 3.45%. The results showed that the physical properties of the simulated maize seeds were similar to those of the real maize seeds. A comparison between the simulated and physical tests is shown in Fig 3 and Fig 4.

### Interparticle contact detection and contact forces

In the EDM, the motion of particles adheres to Newton’s second law. When subjected to external forces, particles undergo displacement changes.‌ Before calculating the forces acting on the particles, contact detection must be performed. ‌Neighbor search is the first step in contact detection, and there are various methods for this, with the cell-linked ‌being one of them.

Due to the non-uniform size of maize particles, when employing the cell-linked‌, the grid cell size was set larger than the longest axis of any maize particle.‌ Maize particles were placed into grid cells based on the positional relationship between the particle model center and the grid cell center. ‌To determine if two maize particles were in contact, a coarse preliminary check was first performed.‌ Using the center of each maize particle as the center and its longest axis as the ‌diameter‌, a ‌bounding sphere‌ was constructed for each particle. The proximity of these two bounding spheres was checked first. ‌If the two large bounding spheres intersected, the component spheres within each maize particle that were potentially in contact with the opposing maize particle’s bounding sphere were identified and flagged. ‌Subsequently, a detailed contact check was performed specifically between these flagged component spheres.‌ If contact was established between the maize particles (meaning their flagged component spheres overlaped), a collision occured, and the ‌overlap amount‌ must be calculated.

Since the maize particles were composed of spherical component spheres, the collision process between maize particles was effectively transformed into collision detection between their component spheres.‌ ‌This collision detection between component spheres was accomplished by comparing the distance D between their centers.‌ ‌If the center-to-center distance D was less than the sum of the radii ($R_{1}+R_{2}$ ) of the two component spheres, then the spheres were in contact.‌ ‌The normal overlap δ at the point of contact was calculated as $\delta =R_{1}+R_{2}-D$. The normal direction was determined by the line connecting the centers of two component spheres.

The Hertz-Mindlin (no slip) contact model employs Hertzian theory to calculate normal forces, while tangential forces are derived from the Mindlin-Deresiewicz formulation. Damping effects are incorporated in both force components, with the damping coefficient linked to the restitution coefficient. Tangential interactions adhere to Coulomb’s friction law, and rolling friction is modeled as a directionally constant torque independent of contact conditions.

In particular, the normal force, *F*_n_ is a function of normal overlap *δ*n and is given by:


$$F_{n}=\frac{\text{4}}{\text{3}}E^{*}\sqrt{R^{*}}{\delta_{n}}^{\frac{\text{3}}{\text{2}}}$$ (1)

$$\frac{\text{1}}{E^{*}}=\frac{\left(1-{v_{i}}^{2}\right)}{E_{i}}+\frac{\left(1-{v_{j}}^{2}\right)}{E_{j}}$$ (2)

$$\frac{\text{1}}{R^{*}}=\frac{\text{1}}{R_{i}}+\frac{\text{1}}{R_{j}}$$ (3)

Where E^*^ is the equivalent Young’s Modulus, Pa;

R^*^ is the equivalent radius, mm.

With E_i_, ν_i_, R_i_, and E_j_, ν_j_, R_j_, being the Young’s Modulus, Poisson ratio and Radius of each sphere in contact. Additionally there is a damping force, F_n_^d^, given by:


$${F_{n}}^{d}=-2\sqrt{\frac{\text{5}}{\text{6}}}\beta \sqrt{S_{n}m^{*}}v_{n}\vec{rel}$$ (4)

$$m^{*}=(\frac{\text{1}}{m_{1}}+\frac{\text{1}}{m_{i}}{)}^{-1}$$ (5)

Where m^*^ is the equiva_l_ent mass, g;

${v_{n}}^{\vec{rel}}$ is the normal component of the relative velocity, m/s;

β and S_n_ (the normal stiffness) are given by:


$$\beta =\frac{-\ln e}{\sqrt{\ln^{2}e+\pi^{2}}}$$ (6)

$$S_{n}=2E^{*}\sqrt{R^{*}\delta_{n}}$$ (7)

With e the coefficient of restitution. The tangential force, F_t_, depends on the tangential overlap δ_t_ and the tangential stiffness S_t_.


$$F_{t}=-S_{t}\delta_{t}$$ (8)

With


$$S_{t}=8G^{*}\sqrt{R^{*}\delta_{n}}$$ (9)

Here G^*^ is the equivalent shear modulus. Additionally, tangential damping is given by:


$${F_{t}}^{d}=-2\sqrt{\frac{\text{5}}{\text{6}}}\beta \sqrt{S_{t}m^{*}}v_{t}\vec{rel}$$ (10)

Where $vn^{\vec{rel}}$ is the relative tangential velocity.

The tangential force is limited by Coulomb friction μ_s_F_n。_

Where μ_s_ is the coefficient of static friction.

For simulations in which rolling friction is important, this is accounted for by applying a torque to the contacting surfaces.


$$\tau_{i}=-\mu_{r}F_{n}R_{i}\omega_{i}$$ (11)

With μ_r_ the coefficient of rolling friction, R_i_ the distance of the contact point from the center of mass and ω_i_, the unit angular velocity vector of the object at the contact point.

### Friction transition trigger criteria and formula expression

When the following criterion is met, the system is in a static friction state:


$$F_{t}\leq \mu_{s}F_{n}$$ (12)

When the following criterion is met, the system transitions from static to kinetic friction:


$$F_{t}>\mu_{s}F_{n}$$ (13)

At this point, the tangential force reaches the maximum static friction limit.

### Calculation of inertia tensor

The maize seed was composed of multiple spherical particles; thus, the inertia tensor of the seed is related to each spherical particle. The calculation formula is as follows:


$$I=\left[\begin{array}{ccc} {}*20cI_{xx} & I_{xy} & I_{xz}\\ I_{yx} & I_{yy} & I_{yz}\\ I_{zx} & I_{zy} & I_{zz} \end{array}\right]$$ (12)

$$I_{xx}=\int (y^{2}+z^{2})dm$$ (13)

$$I_{yy}=\int (x^{2}+z^{2})dm$$ (14)

$$I_{zz}=\int (x^{2}+y^{2})dm$$ (15)

$$I_{xy}=I_{yx}=-\int xydm$$ (16)

$$I_{xz}=I_{zx}=-\int xzdm$$ (17)

$$I_{yz}=I_{zy}=-\int yzdm$$ (18)

Where *I* the inertia tensor of the maize seed relative to an arbitrary fixed point, x, y, and z‌ are the coordinates of spherical particles relative to the fixed point, *I*_xx_, *I*_yy_, and *I*_zz_ moments of inertia, *I*_xy_, *I*_xz_, and *I*_yz_ products of inertia.

### Simulation parameter settings

Three maize seed size categories were established: large-sized (thickness > 8 mm), medium-sized (6 mm ≤ width ≤ 8 mm), and small-sized (width < 6 mm). The weight proportions of large-, medium-, and small-sized seeds were 70%, 20%, and 10%, respectively. Maize seeds were treated as non-cohesive particles, as liquid bridge forces and adhesion effects were considered negligible.

To balance computational accuracy and simulation efficiency, the Hertz-Mindlin no-slip contact model [19] was adopted to characterize inter-seed contact dynamics. The Rayleigh time was set to 25%. During the simulation analysis, the data was saved at an interval of 0.01 s, and the simulation duration was 5 s. The cylinder rotational speed ranged from 24 r/min to 48 r/min, the inclination angle ranged from 0° to 4°, and the feeding rate ranged from 280 g/s to 360 g/s. In the simulation, maize seeds were generated after the grading cylinder had reached stable operation.

**Simulation** **results analysis**

**Fig 5:**
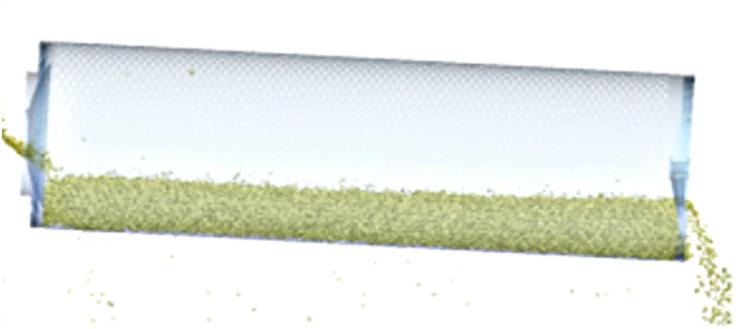
Seed grading model diagram.

**Fig 6:**
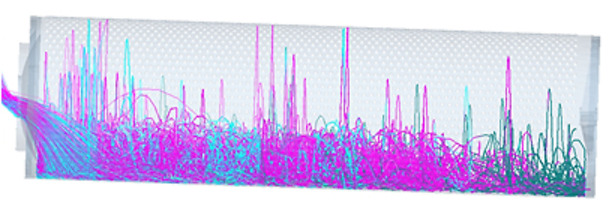
Seed movement trajectory.

Research has demonstrated that the cylinder rotational speed, cylinder inclination angle, and feeding rate are the primary factors influencing the performance of cylindrical seed graders [20]. To investigate the motion and distribution ‌patterns of the maize seeds during cylindrical grading, simulations were conducted by varying these three key parameters. Simulation analysis of maize seed cylinder grading is presented in Fig 5 and Fig 6.

## Analysis of maize seed velocity in the grading cylinder

### Velocity ‌patterns under varying rotational speeds.

**Fig 7:**
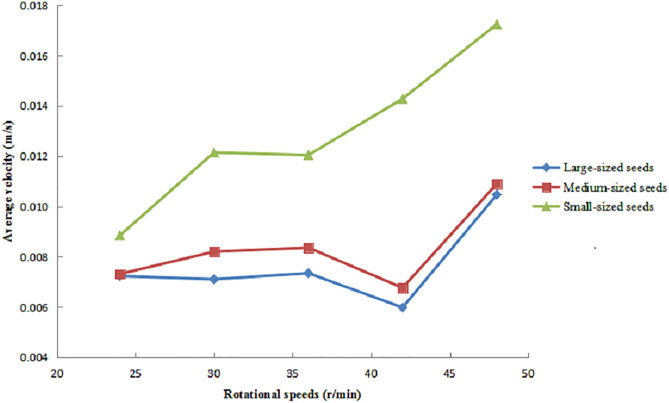
Average circumferential velocity of seeds under different rotational speeds.

The variation in the average circumferential velocity of large-, medium-, and small-sized maize seeds is presented in Fig 7, with rotational speeds ranging from 24 to 48 r/min under a fixed inclination angle of 2° and a feeding rate of 320 g/s. The results showed that as the rotational speed increased, the average circumferential velocity of small-sized seeds exhibited a gradual upward trend and consistently remained higher than that of medium- and large-sized seeds. This increasing trend was most pronounced in the small-sized seeds.

In the range of 24–36 r/min, the circumferential velocity of small-sized seeds increased slowly. However, as the rotational speed increased beyond this range, the velocity rose more rapidly. For medium- and large-sized seeds, the average circumferential velocity showed little change when the rotational speed increased from 24 r/min to 36 r/min. At 42 r/min, their velocities decreased slightly, but at 48 r/min, a significant increase in average circumferential velocity was observed.

**Fig 8:**
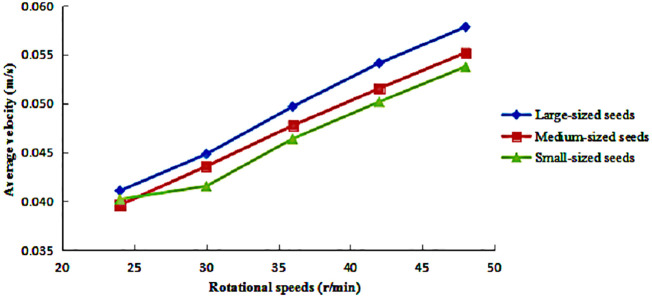
Average axial velocity of seeds under different rotational speeds.

The variation in the average axial velocity of large-, medium-, and small-sized maize seeds under different rotational speeds is illustrated in Fig 8. The results indicate that the average axial velocities of seeds in all size categories exhibited approximately linear increasing trends as the cylinder’s rotational speed increased. The slopes of these trends were remarkably consistent across the different seed sizes. Large-sized seeds maintained the highest axial velocity, followed by medium-sized seeds, while small-sized seeds consistently showed the lowest axial velocity.

### Velocity ‌patterns under varying inclination angles.

**Fig 9:**
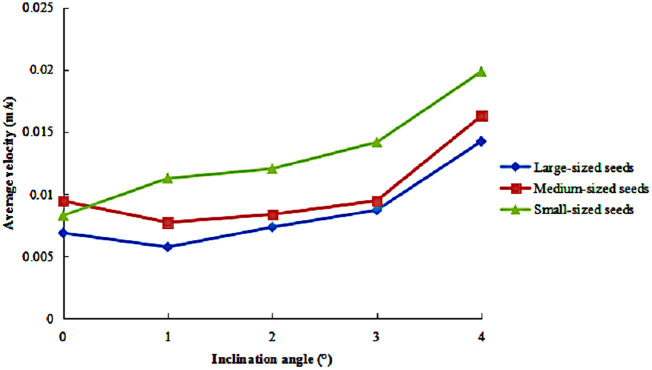
Average circumferential velocity of seeds under different inclination angles.

The variation in the average circumferential velocity of seeds of different sizes is presented in Fig 9, with inclination angles ranging from 0° to 4°, under a fixed rotational speed of 36 r/min and a feeding rate of 320 g/s. The results revealed an overall increasing trend in average circumferential velocity with increasing inclination angle across all seed sizes. Specifically, small-sized seeds exhibited the highest velocity values and showed the most pronounced response to changes in inclination angle, with their velocities increasing continuously. In contrast, medium- and large-sized seeds displayed a characteristic pattern: an initial slight decline in velocity followed by a gradual increase as the inclination angle increased. Throughout all tested conditions, medium-sized seeds consistently maintained higher velocity values than large-sized seeds.

**Fig 10:**
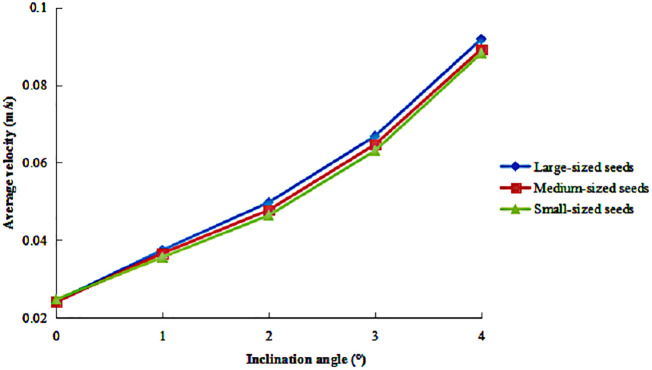
Average axial velocity of seeds under different inclination angles.

The variation in the average axial velocity of seeds of different sizes is presented in Fig 10 under various inclination angles. The results indicated that all seed size fractions exhibited nearly identical increasing trends in axial velocity with increasing inclination angles, with minimal velocity differences observed among them. Comparatively, the amplitude of variation in axial velocity was more sensitive to changes in inclination angle than to changes in rotational speed.

### Velocity ‌patterns under varying feeding rates.

**Fig 11:**
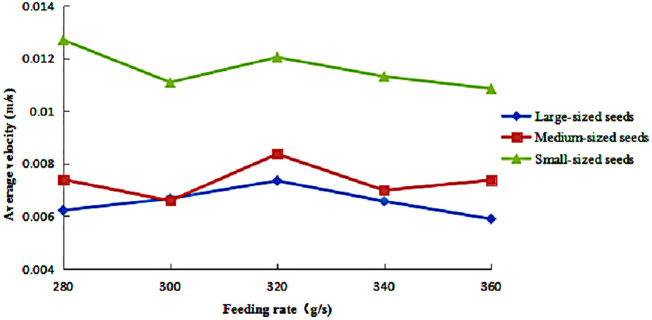
Average circumferential velocity of seeds under different feeding rates.

The variation in the average circumferential velocity of seeds of different sizes is shown in Fig 11, with feeding rates ranging from 280 to 360 g/s under a fixed rotational speed of 36 r/min and an inclination angle of 2°. The analysis revealed distinct velocity variation patterns among the seed size fractions in response to increasing feeding rates. Small-sized seeds exhibited an initial decrease in circumferential velocity, followed by an increase and a subsequent gradual decline, resulting in an overall fluctuating downward trend, while consistently maintaining significantly higher velocities than medium- and large-sized seeds. Medium-sized seeds displayed an oscillatory pattern, characterized by alternating decreases and increases in velocity. Large-sized seeds showed a relatively stable velocity profile, with modest fluctuations that initially increased before declining.

**Fig 12:**
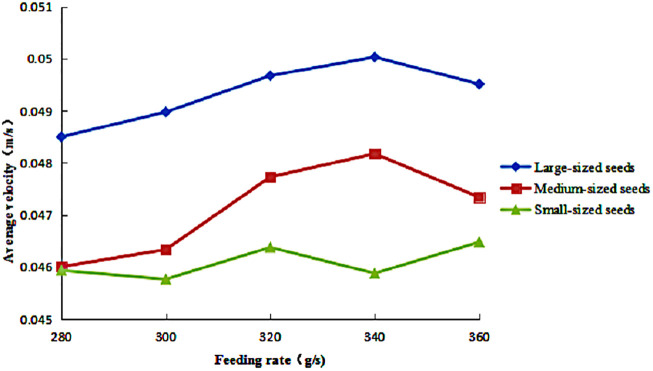
Average axial velocity of seeds under different feeding rates.

The variation in the average axial velocity of seeds of different sizes under different feeding rates is presented in Fig 12. The results demonstrated distinct size-dependent behavioral patterns. For medium- and large-sized seeds, axial velocity initially increased with feeding rate, then decreased, forming a characteristic unimodal response. Notably, medium-sized seeds exhibited greater amplitude in velocity variation than large-sized seeds, although they consistently maintained lower absolute velocities across the tested range. In contrast, small-sized seeds displayed a fundamentally different response, with a fluctuating yet overall increasing trend in axial velocity as the feeding rate increased. Small-sized seeds consistently recorded the lowest axial velocities among all size fractions

## Analysis of maize seed distribution in the grading cylinder

The distribution heterogeneity of seed particles within the cylinder was quantitatively characterized using the coefficient of variation (CV), where a lower CV value indicated better distribution uniformity. The CV‌ is the ratio of the standard deviation to the mean, eliminating the influence of units and enabling the comparison of the dispersion across different datasets. The ‌formula‌ is as follows:


$$c_{v}=\frac{\sigma }{\mu }$$ (19)

where c_v_ is the ‌coefficient of variation;

*σ* is the ‌standard deviation‌ of the data (indicating dispersion);

*μ* is the ‌mean‌ of the data (indicating central tendency).

### Distribution uniformity under varying rotational speeds.

**Fig 13:**
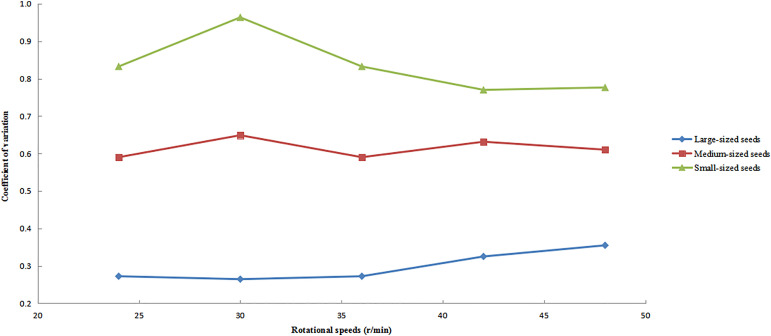
Circumferential CV of seeds under different rotational speeds.

The variation patterns of the circumferential CV for large-, medium-, and small-sized seeds at different rotational speeds are shown in Fig 13, with all other operational parameters maintained as previously specified. The analysis revealed distinct response patterns to changes in rotational speed among the different seed size fractions. Large-sized seeds consistently exhibited the lowest CV values and showed a monotonically increasing trend. Medium-sized seeds demonstrated the most stable performance, with minimal CV fluctuations. In contrast, small-sized seeds displayed the greatest variability, characterized by an initial increase in CV followed by a subsequent decrease, resulting in the largest amplitude of variation among all size categories. This was because large-sized seeds had greater mass and stronger inertial effects, enabling them to maintain more stable trajectories at low speeds with higher initial distribution uniformity (lower CV). As rotational speed increased, centrifugal forces intensified. Due to their greater mass, large-sized seeds struggled to rapidly adjust positions, leading to gradual accumulation of slight deviations and a slow rise in CV. Additionally, large-sized seeds had larger contact areas with the cylinder wall, resulting in significant frictional resistance. At higher speeds, sliding friction effects became more pronounced, further hindering uniform distribution. Small-sized seeds, with their lesser mass and weaker inertia, were more susceptible to collisions and friction during the grading process. At low speeds, random motion dominated (causing CV to rise), while at high speeds, the guiding effect of centrifugal force strengthened (leading to a decrease in CV). Similarly, when other key factors (such as inclination angle and feeding rate) changed, large seeds exhibited the smallest CV, with overall fluctuations being smaller compared to small seeds, resulting in a more stable distribution state.

**Fig 14:**
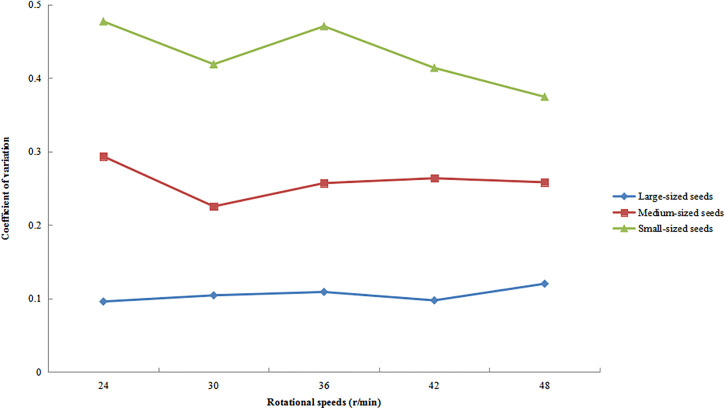
Axial CV of seeds under different rotational speeds.

The variation patterns of the axial CV for large-, medium-, and small-sized seeds at different rotational speeds are presented in Fig 14. The results indicated that small seeds exhibited a general decreasing trend in CV with increasing rotational speed, although they consistently maintained higher variability than medium- and large-sized seeds. Both medium- and large-sized seeds showed relatively stable CV responses to changes in rotational speed, with minimal fluctuations. Notably, medium-sized seeds consistently demonstrated higher CV values than large-sized seeds, while large-sized seeds maintained the lowest CV magnitudes throughout the test conditions, indicating the most consistent axial movement patterns among all size classes.

### Distribution uniformity under varying inclination angles.

**Fig 15:**
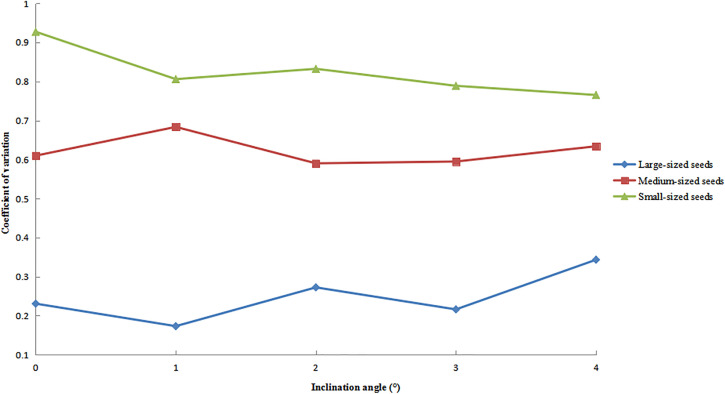
Circumferential CV of seeds under different inclination angles.

The variation patterns of the circumferential CV for large-, medium-, and small-sized seeds at different inclination angles are shown in Fig 15, with all other operational parameters maintained as previously specified. The results indicated that the amplitude of CV variation with increasing inclination angle was comparable across the different seed size categories. Among them, small-sized seeds exhibited the highest CV values, showing a gradually decreasing trend. Medium-sized seeds displayed intermediate CV values, with an initial increase followed by a decrease, eventually stabilizing. Large-sized seeds consistently maintained the lowest CV values and exhibited oscillatory fluctuations accompanied by a slow upward trend.

**Fig 16:**
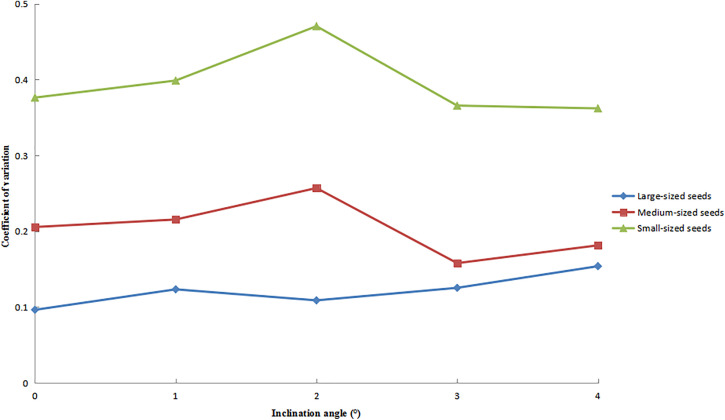
Axial CV of seeds under different inclination angles.

The variation patterns of the axial CV for large-, medium-, and small-sized seeds at different inclination angles are shown in Fig 16. The results demonstrated that, with increasing inclination angle, small-sized seeds initially exhibited an increase in CV values, followed by a decrease, while consistently maintaining the highest CV among all size fractions. Medium-sized seeds showed a similar trend. In contrast, large-sized seeds exhibited a gradually increasing trend in CV values but consistently maintained the lowest CV values across all test conditions.

### Distribution uniformity under varying feeding rates.

**Fig 17:**
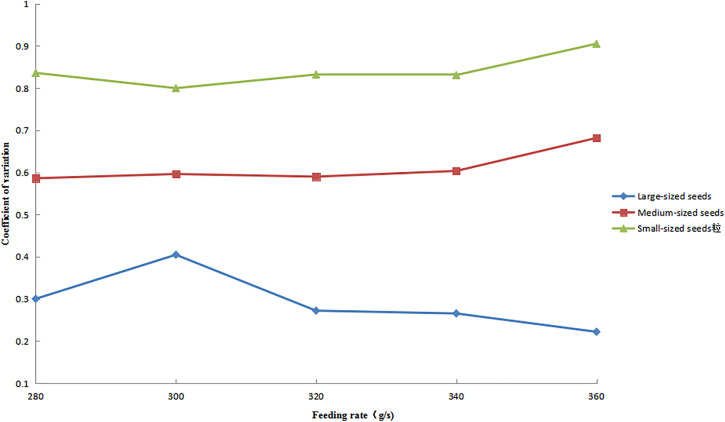
Circumferential CV of seeds under different feeding rates.

The variation patterns of circumferential CV for large-, medium-, and small-sized seeds under different feeding rates are shown in Fig 17, with all other operational parameters maintained as previously specified. The results showed that small- and medium-sized seeds exhibited similar variation trends, with their CV values gradually increasing and then remaining relatively stable as the feeding rate increased. Notably, the CV values of small-sized seeds consistently exceeded those of medium-sized seeds throughout the experiment. In contrast, large-sized seeds showed an initial increase in CV values, followed by a decrease. Although large seeds maintained the lowest CV values overall, they displayed the greatest amplitude of variation among all seed size fractions.

**Fig 18:**
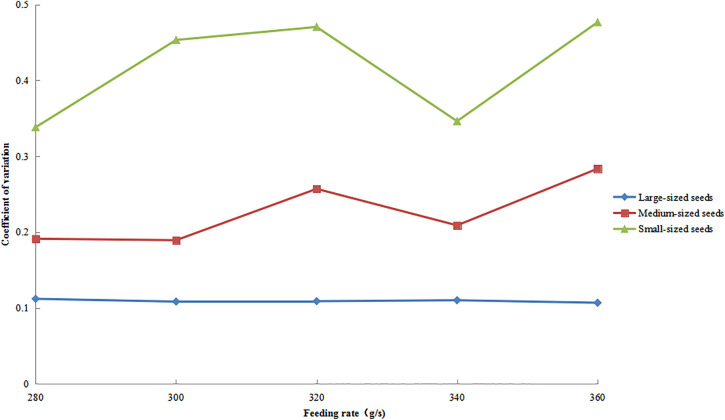
Axial CV of seeds under different feeding rates.

The variation patterns of the axial CV for large-, medium-, and small-sized seeds under different feeding rates are shown in Fig 18. The results revealed that both small- and medium-sized seeds exhibited a characteristic pattern of an initial increase, subsequent decrease, and final increase in CV values as feeding rates rose. Throughout this process, small-sized seeds consistently showed higher CV values and greater variation amplitudes than medium-sized seeds. Medium-sized seeds demonstrated an overall fluctuating upward trend in CV values. In contrast, large-sized seeds maintained the lowest and most stable CV values, with minimal variation amplitude across all feeding rate conditions.

## Experiment

In the process of cylindrical grading, seed grading quality was significantly improved when maize seeds exhibited a higher contact probability with the separation cylinder, longer contact duration, and a more uniform seed distribution. Based on the analysis of maize seed motion and distribution patterns, orthogonal experiments were conducted using EDEM 2018 simulation software to identify the optimal parameter combination for achieving superior grading quality. By comparing simulation results with bench test data obtained under the optimal parameter combination, the validity of the motion and distribution pattern simulations, as well as the accuracy of the optimal parameters derived from the orthogonal experiments, were effectively confirmed.

### Box-Behnken experiment

A response surface methodology study was implemented through simulation analysis, with the grading qualification rate (*G*) as the response variable and rotational speed (*A*), inclination angle (*B*), and feeding rate (*C*) as the influencing factors. A three-factor quadratic regression experimental design [21] was employed to determine the optimal combination of key parameters affecting grading efficiency. Based on simulation analysis, the determined ranges for each parameter were as follows: the cylinder rotational speed ranged from 36 r/min to 48 r/min, the inclination angle ranged from 0° to 2°, and the feeding rate ranged from 300 g/s to 340 g/s. The experimental factors and their corresponding levels are listed in S4 Table.

The experiment was conducted using Design-Expert software version 8.0.6.1, applying the Box-Behnken design method. The design included five replicates and 17 experimental runs. The test design and results are presented in S5 Table.

### Regression model establishment and significance test

Based on the test results, multiple regression fitting was performed using Design-Expert 8.0.6 software to develop a regression model for the grading qualification rate *G* as a function of the three independent variables: rotational speed *A*, inclination angle *B*, and feeding rate *C*, as shown in Equation (20). The results of the variance analysis for the regression model are summarized in S6 Table.


$$G=95.22+0.6A-5.11B-0.095C+0.93AB+0.11AC-0.018BC+0.27A^{2}-4.09B^{2}+0.26C^{2}$$ (20)

As shown in S6 Table, the qualification rate model demonstrated high statistical significance (*P* < 0.01) and a good fit to the regression equation (*P* > 0.05 for lack of fit). The coefficient of determination (*R²*) of 0.993, indicating a strong correlation between the predicted and actual values, with minimal experimental error. This confirms the model’s suitability for analyzing and predicting the grading qualification rate *G* of maize seeds.

Among the factors examined, the inclination angle *B* and its quadratic term *B²* had highly significant effects (*P* < 0.01), while rotational speed *A* and the interaction term *AB* showed significant influences (*P* < 0.05). The remaining regression terms were statistically insignificant (*P* > 0.05). According to the regression coefficient analysis [22,23], the relative importance of the factors affecting the qualification rate ranked as follows: inclination angle > rotational speed > feeding rate.

### Response surface analysis

**Fig 19:**
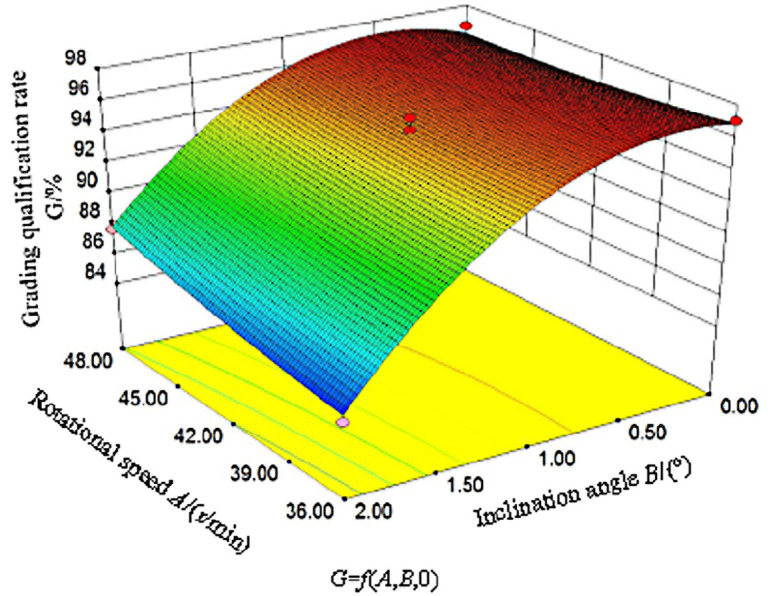
Interactive effects of experimental factors on grading qualification rate.

Fig 19 shows the response surface plot of the rotational speed and inclination angle at the central level of the feeding rate (320 g/s), generated using Design-Expert 8.0.6. The results indicated that the grading qualification rate initially increased and then slightly decreased as the cylinder inclination angle decreased. This trend can be attributed to the reduction in the axial velocity of the maize seeds with a decreasing inclination angle, which prolonged their residence time from the feeding inlet to the discharge outlet. Consequently, the interaction duration and probability between the seeds and the grading cylinder increased. Additionally, the uniformity of seed distribution in both the circumferential and axial directions improved, leading to an enhanced grading qualification rate. However, when the inclination angle decreased further, the discharge rate of the seeds gradually decreased. When the feeding rate substantially exceeded the discharge rate, excessive seed accumulation in the cylinder reduced the contact probability between the seeds and the cylinder surface, resulting in a gradual decrease in the grading qualification rate.

The variation in the grading qualification rate with cylinder rotational speed was relatively moderate. At high inclination angles, the grading qualification rate increased with rotational speed owing to the enhanced circumferential velocity of the seeds, which improved their contact frequency and duration with the cylinder. The improved distribution uniformity in both the circumferential and axial directions also contributed to higher grading performance. Conversely, at low inclination angles, the increased seed population within the cylinder counteracted the positive effects of the elevated axial velocity and improved distribution uniformity, thereby diminishing the influence of rotational speed on the grading qualification rate. Under these conditions, the grading qualification rate exhibited minimal variation with rotational speed.

### Parameter optimization

With the objective of maximizing the grading qualification rate (*G*), the derived regression model was optimized under the constraints specified in Equation (21) to maximize the grading qualification rate *(G)*. The optimal solution was determined as follows: rotational speed of 47.08 r/min, inclination angle of 0.52°, and feeding rate of 303.07 g/s. Under these conditions, the predicted maximum grading qualification rate (*G*) reached 97.24%.


$$\left\{ \begin{array}{c} \max G(A,B,C)\\ -1\leq A\leq 1\\ -1\leq B\leq 1\\ -1\leq C\leq 1 \end{array}\right.$$ (21)

### Validation experiment

A cylindrical seed grading experimental setup was constructed to verify the simulated grading qualification rate. Based on simulation results and practical considerations, the optimal operational parameters were established as follows: cylinder rotational speed of 47 r/min, inclination angle of 0.5°, and feeding rate of 303 g/s. The experimental procedures required initiating seed feeding only after achieving stable operation of the grading cylinder. The grading qualification rate was adopted as the primary evaluation metric according to NY/T 366–2020 “Technical Specification of Quality Evaluation for Seed Grader” [24], with the calculation method defined by Equation (22). This standardized approach ensured the reliability and comparability of the experimental results.


$$G=\frac{M_{1}}{M_{2}}$$ (22)

where *G* is the grading qualification rate, %;

M_1_ is the mass of qualified seeds collected from the primary outlet, g;

M_2_ is the total mass of sample collected from the primary outlet, g.

Five replicate trials were conducted [25–27], with the average grading qualification rate calculated from the results. As shown in S7 Table, the validation experiments demonstrate a relative error of less than 4% compared with the simulation results. This close agreement confirms both the validity of the motion and distribution pattern simulation analyses and the accuracy of the optimal parameter combination obtained through the orthogonal experimental design.

## Discussion

The mixing of particles within a cylinder is generally considered to result from the combined effects of three mixing mechanisms: convective, diffusive, and shear. Studies [28–31] indicate that significant segregation and stratification phenomena occur during the mixing of particles of different sizes, with up to 13 segregation mechanisms having been summarized [32]. Since the maize seed population to be graded consists of particles with varying morphological dimensions, the cylindrical grading process involves a highly complex coupling of competing mixing and segregation mechanisms. This study employed EDEM 2018 simulation to investigate the motion and distribution patterns of seeds under different operational parameters during cylindrical grading, which helps improve the performance of the grading machine. Additionally, this research provides theoretical and methodological references for analyzing the motion and distribution patterns of other granular materials in rotating cylinders.

In this study, the motion and distribution patterns of maize seeds of different sizes were obtained using simulation analysis software. Experimental verification is required to validate the rationality of the simulation. However, in the verification experiments, due to the large number of seed particles inside the cylinder, the internal maize particles were obscured and could not be directly observed. This made it impossible to directly compare the average velocity and CV of the seeds between the simulation and experimental results. Therefore, this study indirectly validates the rationality of the simulation analysis of maize seed motion by comparing the grading qualification rates between the simulation and experimental tests, thereby confirming the accuracy of the optimal parameter combination obtained from the orthogonal experiments.

This study has certain limitations. Various factors such as seed variety, shape, moisture content, and density may influence the motion patterns of maize seeds. Due to experimental constraints, these factors were not considered in this study. Future research should focus on these variables to further refine the theoretical framework.

## Conclusion

In this study, discrete element models of maize seeds and the grading cylinder were developed using EDEM 2018 software to simulate and analyze the variation patterns of circumferential and axial motion velocities, as well as the CV of maize seeds under different rotational speeds, inclination angles, and feeding rates. Additionally, the Box-Behnken central composite design method was employed, and Design-Expert software was used to determine the order of significance of each factor affecting the grading qualification rate: inclination angle, rotational speed, and feeding rate. The interaction between inclination angle and rotational speed had a significant effect on the grading qualification rate, whereas the interactions between other factors did not show significant effects. The optimal parameter combination obtained from the simulation was as follows: rotational speed of 47.08 r/min, inclination angle of 0.52°, and feeding rate of 303.07 g/s, with a predicted grading qualification rate of 97.24%. Validation experiments were conducted using this optimal parameter combination, with the grading qualification rate as the performance indicator. The experimental grading qualification rate was 93.83%, and the relative error between the experimental and predicted values was less than 4%, confirming the rationality of the simulation analysis of maize seed motion behavior.

## Supporting information

S1 Table Parameter of maize seed of DEM. The parameters of maize seeds can be measured using specialized instruments or obtained from the relevant literature.
(XLSX)

S2 Table Morphological dimensional parameters of maize seeds. The dimensional parameters of the seeds were measured using a vernier caliper.
(XLSX)

S3 Table Parameter of grading cylinder of DEM. The parameters of the grading cylinder were obtained from the materials manual.
(XLSX)

S4 Table Response surface test factors and levels. The table lists three levels of parameter setting for the three factors in the test.
(XLSX)

S5 Table Test design and results. The table lists all 17 experimental points.
(XLSX)

S6 Table Variance analysis of the regression equation. Note: P < 0.01 (highly significant, **); 0.01 ≤ P < 0.05 (significant, *).
(XLSX)

S7 Table Comparison between predicted values and validation test results. (XLSX)
